# Association between baseline LDL-C and prognosis among patients with coronary artery disease and advanced kidney disease

**DOI:** 10.1186/s12882-021-02375-1

**Published:** 2021-05-06

**Authors:** Bo Wang, Shiqun Chen, Jin Liu, Yan Liang, Liangguang Meng, Xiaoming Yan, Haozhang Huang, Guanzhong Chen, Zhidong Huang, Danyuan Xu, Min Li, Jingjing Liang, Shuangxin Liu, Jiyan Chen, Yong Liu, Ning Tan

**Affiliations:** 1 Department of Cardiology, Guangdong Provincial Key Laboratory of Coronary Heart Disease Prevention, Guangdong Cardiovascular Institute, Guangdong Provincial People’s Hospital, Guangdong Academy of Medical Sciences, Guangzhou, 510080 China; 2 Maoming People’s Hospital, Maoming, 525000 China; 3 Department of Information Technology, Guangdong Provincial People’s Hospital, Guangdong Academy of Medical Sciences, Guangzhou, 510080 China; 4 The Second School of Clinical Medicine, Southern Medical University, Guangzhou, 510515 China; 5 Guangdong Provincial People’s Hospital, School of Medicine, South China University of Technology, Guangzhou, 510100 China; 6 Division of Nephrology, Guangdong Provincial People’s Hospital, Guangdong Academy of Medical Sciences, Guangzhou, 510080 China

**Keywords:** Baseline low-density lipoprotein cholesterol • coronary artery disease • advanced kidney disease • all-cause mortality

## Abstract

**Background:**

Lower low-density lipoprotein cholesterol (LDL-C) is significantly associated with improved prognosis in patients with coronary artery disease (CAD). However, LDL-C reduction does not decrease all-cause mortality among CAD patients when renal function impairs. The association between low baseline LDL-C (< 1.8 mmol/L) and mortality is unknown among patients with CAD and advanced kidney disease (AKD). The current study aimed to evaluate prognostic value of low baseline LDL-C level for all-cause death in these patients.

**Methods:**

In this observational study, 803 CAD patients complicated with AKD (eGFR < 30 mL/min/1.73 m^2^) were enrolled between January 2008 to December 2018. Patients were divided into two groups (LDL-C < 1.8 mmol/L, *n* = 138; LDL-C ≥ 1.8 mmol/L, *n* = 665). We used Kaplan-Meier methods and Cox regression analyses to assess the association between baseline low LDL-C levels and long-term all-cause mortality.

**Results:**

Among 803 participants (mean age 67.4 years; 68.5% male), there were 315 incidents of all-cause death during a median follow-up of 2.7 years. Kaplan–Meier analysis showed that low LDL-C levels were associated with worse prognosis. After adjusting for full 24 confounders (e.g., age, diabetes, heart failure, and dialysis, etc.), multivariate Cox regression analysis revealed that lower LDL-C level (< 1.8 mmol/L) was significantly associated with higher risk of all-cause death (adjusted HR, 1.38; 95% CI, 1.01–1.89).

**Conclusions:**

Our data demonstrated that among patients with CAD and AKD, a lower baseline LDL-C level (< 1.8 mmol/L) did not present a higher survival rate but was related to a worse prognosis, suggesting a cautiousness of too low LDL-C levels among patients with CAD and AKD.

**Supplementary Information:**

The online version contains supplementary material available at 10.1186/s12882-021-02375-1.

## Introduction

The efficacy of the low-density lipoprotein cholesterol (LDL-C) reduction can ameliorate the cardiovascular mortality of patients with coronary artery disease (CAD) [1–7]. For patients with CAD and advanced kidney disease (AKD, defined as an eGFR below 30 mL/min/1.73 m2), ISCHEMIA-CKD study recommended a criterion of optimal medical therapy that LDL-C should be controlled below1.8 mmol/L [8]. A systematic review and meta-analysis conducted by the Cholesterol Treatment Trialists (CTT) and the SHARP trial suggested that in patients with poor renal function would not result in an improvement of clinical outcomes [9, 10]. Kovesdy et al.’s study also found that lower LDL-C levels were associated with higher mortality in patients who have moderate and advanced CKD and are not yet on dialysis [11].

However, for patients with CAD and AKD (eGFR < 30 mL/min/1.73 m2), the association between low baseline LDL-C (< 1.8 mmol/L) and mortality is unknown. Therefore, the present study aimed to investigate the lower baseline LDL-C value in terms of their prognostic value for all-cause death in patients with CAD and AKD.

## Method

### Study design and participants

**Fig. 1:**
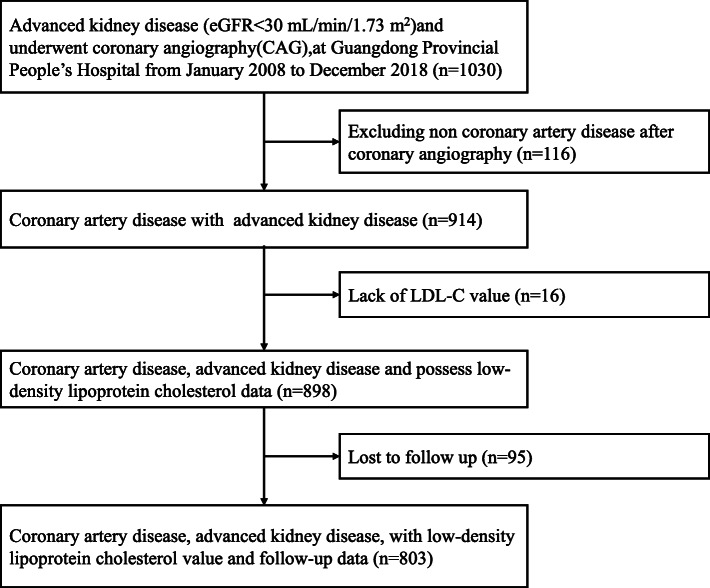
Study flow chart

This observational study was conducted at Guangdong Provincial People’s Hospital in China. From January 2008 to December 2018 and was firstly registered with ClinicalTrials.gov on 29th May 2020 (NCT04407936). We retrospectively enrolled 1030 consecutive patients complicated with AKD (eGFR<30 mL/ min/1.73 m^2^) and undergoing coronary angiography (CAG). We excluded 116 patients without CAD, 16 patients who did not test for LDL-C, and 95 patients who were lost to follow-up. Finally, 803 patients were included in this analysis (see Fig. 1). The study protocol was approved by the Guangdong Provincial People’s Hospital ethics committee, and the study was performed according to the Declaration of Helsinki.

### Procedures

At enrollment, we collected baseline data including demographic characteristics, coexisting conditions, laboratory examinations, and medications. CAG or percutaneous coronary intervention (PCI) was performed in accordance with standard clinical practice guidelines [12–14].

### Clinical outcome and definition

AKD was defined as an estimated glomerular filtration rate (eGFR) < 30 mL/ (min·1.73 m^2^), which was calculated by the Modification of Diet in Renal Disease (MDRD) formula [15]. CAD was confirmed by CAG. LDL-C value was measured by overnight fasting blood sample and used the first test result during hospitalization. Congestive heart failure (CHF) was defined as New York Heart Association (NYHA) class > 2 or Killip class > 1. Hypertension was definitively present if the participant was under treatment with antihypertensive medication or had systolic blood pressure ≥ 140 mmHg or diastolic blood pressure ≥ 90 mmHg [16]. Follow-up data were monitored and recorded by trained nurses through outpatient interviews and telephones.

### Statistical analysis

Descriptive statistics are reported as the mean ± standard deviation (SD), median (interquartile range [IQR]), or number and percentage when appropriate. The chi-square test was used to compare differences between categorical variables. An independent-samples Student’s t-test was used to compare continuous variables with normal distribution, and the Wilcoxon rank sum test was used to compare continuous variables. Analysis of the rate of death from any cause was performed with the use of Kaplan-Meier methods, with survival curves for the probability of remaining outcome-free in the two groups divided by LDL-C level of 1.8 mmol/L. The two curves were compared with the method of the log-rank test. Cox proportional hazards models were used to investigate the associations of baseline LDL-C levels with long-term all-cause death. The following six multivariate Cox models were sequentially constructed with different covariates: (1) adjusted for age and male; (2) adjusted for nutritional status: albumin; (3) adjusted for lipid variables: total cholesterol, triglyceride, high-density lipoprotein cholesterol; (4) adjusted for variables associated with cardiovascular disease: acute coronary syndrome, congestive heart failure, hypertension, peripheral arterial disease, previous acute myocardial infarction, PCI, Lg ProBNP; (5) adjusted for variables associated with kidney disease: dialysis, 15 ≤ eGFR < 30 ml/min/1.73 m^2^ vs. eGFR < 15 ml/min/1.73 m^2^; (6) adjusted for full multivariate variables: age, male, current smoker, acute coronary syndrome, congestive heart failure, hypertension, diabetes mellitus, dialysis, anemia, peripheral arterial disease, 15 ≤ eGFR < 30 ml/min/1.73 m^2^ vs. eGFR < 15 ml/min/1.73 m^2^, previous acute myocardial infarction, PCI, white blood cell count, albumin, total cholesterol, triglyceride, high-density lipoprotein cholesterol, Lg ProBNP, Lg D-dimer, ACEI/ARB, β-blockers, statins, diuretics. The hazard ratio (HR) and 95% confidence interval (CI) were calculated. We performed multiple imputations using Markov chain Monte Carlo (MCMC) and fully conditional specification (FCS) to avoid exclusion of patients with missing values. 5 variables were conducted by nonparametric multiple imputation with missing-at-random assumptions [17, 18]. Patients who had missing predictors over 20% were excluded. Variables were significant at *P* < 0.05 according to univariate Cox regression, and those associated with mortality according to clinical experience were further controlled by multivariable Cox regression in 6 different models. Model 1 adjusted age and gender. Model 2 adjusted nutritional status (albumin). Mode 3 adjusted 3 lipid variables (total cholesterol, triglyceride, high density lipoprotein cholesterol). Model 4 adjusted for variables associated with cardiovascular disease. Model 5 adjusted for variables associated with kidney disease. And model 6 adjusted for full multivariate variables. We also performed sensitivity analysis, including comparing the differences before and after data filling and subgroup analysis. Potential nonlinear associations between the concentration of LDL-C and long-term all-cause mortality were examined with restricted cubic splines. All data analyses were performed using SAS, version 9.4 (SAS Institute, Cary, NC) and R software, version 4.0.1 (R Foundation for Statistical Computing). All *p* values < 0.05 were considered to represent statistical significance.

## Result

From January 2008 to December 2018, a total of 803 patients were included in the study. The mean age was 67.4 ± 10.3 years, and 550 (68.5%) were male. There was 312 (38.9%) death during a median follow-up of 2.7 (IQR 1.6–4.4) years. A total of 803 eligible patients were divided into a low LDL-C group (*n* = 138) and a high LDL-C group (*n* = 665). All of the patients’ baseline clinical characteristics are shown in Table 1.

**Table 1:** Baseline characteristics

Characteristic^a^	Missing Data	Overall (*N* = 803)	LDL-C ≥ 1.8 mmol/L(*N* = 665)	LDL-C < 1.8 mmol/L(*N* = 138)	*P* value
**Demographic characteristics**					
Age, year	0 (0)	67.4 (10.3)	67.5 (10.2)	66.8 (10.8)	0.44
Male, n (%)	0 (0)	550 (68.5)	450 (67.7)	100 (72.5)	0.32
**Coexisting conditions**					
Current smoker, n (%)	0 (0)	117 (14.6)	99 (14.9)	18 (13.0)	0.67
ACS, n (%)	0 (0)	386 (48.1)	328 (49.3)	58 (42.0)	0.14
CHF, n (%)	0 (0)	381 (47.4)	312 (46.9)	69 (50.0)	0.57
Hypertension, n (%)	0 (0)	654 (81.4)	530 (79.7)	124 (89.9)	0.008
Diabetes mellitus, n (%)	0 (0)	429 (53.4)	343 (51.6)	86 (62.3)	0.03
Dialysis, n (%)	0 (0)	283 (35.2)	216 (32.5)	67 (48.6)	< 0.001
Anemia, n (%)	2 (0.2)	662 (82.6)	537 (80.9)	125 (91.2)	0.005
PAD, n (%)	0 (0)	91 (11.3)	80 (12.0)	11 (8.0)	0.22
eGFR, ml/min/1.73 m^2^	0 (0)	18.0 (8.3)	18.4 (8.1)	15.9 (8.9)	0.001
15 ≤ eGFR < 30 ml/min/1.73 m^2^, n (%)	0 (0)	499 (62.1)	431 (64.8)	68 (49.3)	0.001
Previous AMI, n (%)	0 (0)	64 (8.0)	54 (8.1)	10 (7.2)	0.86
PCI, n (%)	0 (0)	440 (54.8)	365 (54.9)	75 (54.3)	0.98
**Laboratory examination**					
WBC, 10^9^/L	2 (0.2)	8.6 (3.3)	8.6 (3.3)	8.5 (3.7)	0.67
ALB, g/L	1 (0.1)	32.7 (5.1)	32.9 (5.2)	32.0 (4.5)	0.06
Total Cholesterol, mmol/L	1 (0.1)	4.4 (1.3)	4.6 (1.3)	3.2 (0.8)	< 0.001
LDLC, mmol/L	0 (0)	2.7 (1.0)	2.9 (0.9)	1.5 (0.3)	< 0.001
TRIG, mmol/L	0 (0)	1.3 (1.36)	2.0 (1.3)	1.8 (1.4)	0.09
HDLC, mmol/L	0 (0)	0.9 (0.3)	0.9 (0.3)	0.8 (0.2)	< 0.001
Lg ProBNP, pg/mL	113 (14.1)	3.7 (0.7)	3.7 (0.7)	3.8 (0.7)	0.18
Lg D-dimer, ng/mL	42 (5.2)	3.1 (0.4)	3.1 (0.4)	3.0 (0.4)	0.71
**Medications**					
ACEI/ARB, n (%)	0 (0)	329 (41.0)	268 (40.3)	61 (44.2)	0.45
β-blockers, n (%)	0 (0)	542 (67.5)	453 (68.1)	89 (64.5)	0.47
Statins, n (%)	0 (0)	717 (89.3)	593 (89.2)	124 (89.9)	0.93
Diuretics, n (%)	0 (0)	337 (42.0)	281 (42.3)	56 (40.6)	0.79
**Events**					
Death, n (%)	0 (0)	312 (38.9)	238 (35.8)	74 (53.6)	< 0.001

^a^ Data are presented as the mean value standard deviation or percentage of participants

Abbreviations: *LDL-C* Low-density lipoprotein cholesterol; *ACS* Acute coronary syndrome; *CHF* Congestive heart failure; *PAD* Peripheral arterial disease; *eGFR* Estimated glomerular filtration rate; *AMI* Acute myocardial infarction; *PCI* Percutaneous coronary intervention; *HDL-C* High-density lipoprotein cholesterol; *TRIG* Triglycerides; *WBC* White blood cell; *pro-BNP* Pro-brain natriuretic peptide; *ACEI* Angiotensin-Converting Enzyme Inhibitors; *ARB* Angiotensin Receptor Blockers

Generally, participants with low LDL-C levels had lower HDL-C levels. In the low LDL-C group, patients were more likely to have diabetes mellitus (62.3% vs 51.6%, *p* = 0.03), hypertension (89.9% vs 79.7%, *p* = 0.008), and anemia (91.2% vs 80.9%, *p* = 0.005) and to be undergoing dialysis (62.3% vs 51.6%, *p* < 0.001). The proportions of male, current smoker, CHF, peripheral arterial disease (PAD), previous acute myocardial infarction (AMI), PCI and medications were similar in both groups. Based on the comparison of complete data and missing data, data are missing at random (Supplementary Table 1).

**Fig. 2:**
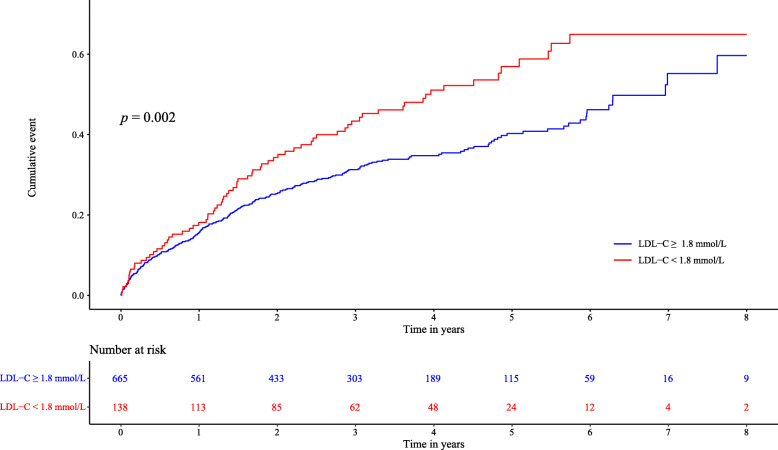
Kaplan-Meier curve of long-term mortality according to LDL-C levels

Kaplan-Meier survival analysis demonstrated that patients in the low LDL-C group had a significantly higher long-term all-cause mortality than those in the high LDL-C group (log-rank *P* = 0.002; Fig. 2).

Univariate regression analysis showed that lower baseline LDL-C level was significantly associated with the primary endpoint (Supplementary Table 2). Potential predictive factors for all-cause long-term mortality were adjusted using multivariable Cox regression analysis. Adjusted for full multivariate variables (including 24 confounders such as age, diabetes, heart failure, and dialysis, etc), baseline LDL-C < 1.8 mmol/L was associated with all-cause long-term mortality (HR: 1.38, 95% CI: 1.01–1.89, *p* = 0.04). and other five models have similar results (see Table 2).

**Table 2:** Cox proportional hazard ratios of LDL-C < 1.8 mmol/L vs. LDL-C ≥ 1.8 mmol/L for long-term all-cause mortality in different models

	HR	95% CI		*P* Value
Univariate Cox model	1.53	1.18	1.99	0.001
Model 1	1.52	1.17	1.98	0.002
Model 2	1.47	1.13	1.91	0.004
Model 3	1.58	1.16	2.14	0.003
Model 4	1.40	1.08	1.83	0.012
Model 5	1.45	1.12	1.88	0.005
Model 6	1.38	1.01	1.89	0.042

Model 1: Adjusted for age and male

Model 2: Adjusted for nutritional status: albumin

Model 3: Adjusted for lipid variables: total cholesterol, triglyceride, high-density lipoprotein cholesterol

Model 4: Adjusted for variables associated with cardiovascular disease: ACS, CHF, hypertension, PAD, previous AMI, PCI, Lg ProBNP

Model 5: Adjusted for variables associated with kidney disease: dialysis, 15 ≤ eGFR < 30 ml/min/1.73 m^2^ vs. eGFR < 15 ml/min/1.73 m^2^

Model 6: Adjusted for full multivariate variables: age, male, current smoker, ACS, CHF, hypertension, diabetes mellitus, dialysis, anemia, PAD, 15 ≤ eGFR < 30 ml/min/1.73 m^2^ vs. eGFR < 15 ml/min/1.73 m^2^, previous AMI, PCI, WBC, albumin, total Cholesterol, triglyceride, high-density lipoprotein cholesterol, Lg ProBNP, Lg D-dimer, ACEI/ARB, β-blockers, statins, diuretics

Subgroup analyses were conducted by dialysis or not, ACS, and diabetes mellitus (DM) (see Table 3). In the subgroup analysis of long-term all-cause mortality among patients undergoing dialysis and with DM, the relation was similar (HR: 1.66, 95% CI: 1.03–2.68, p = 0.04; HR: 1.59, 95% CI: 1.05–2.40, *p* = 0.03).

**Table 3:** Subgroup analysis of LDL-C level with mortality among CAD and CKD patients

Subgroup	No of events / No of patients		Multivariate Cox Regression				P for interaction
	LDL-C < 1.8	LDL-C ≥ 1.8	HR	95% CI		*P* value	
On dialysis							
Yes	39/67 (58.2%)	86/216 (39.8%)	1.66	1.03	2.68	0.04	0.38
No	36/71 (50.7%)	154/449 (34.3%)	1.22	0.79	1.88	0.38	
ACS							
Yes	31/58 (53.4%)	120/328 (36.6%)	1.36	0.84	2.19	0.21	0.76
No	44/80 (55.0%)	120/337 (35.6%)	1.33	0.87	2.02	0.18	
DM							
Yes	48/86 (55.8%)	129/343 (37.6%)	1.59	1.05	2.40	0.03	0.35
No	27/52 (51.9%)	111/322 (34.5%)	1.30	0.78	2.18	0.32	

Abbreviations: *ACS* Acute coronary syndrome; *DM* Diabetes mellitus

Adjusted for full multivariate variables: age, male, current smoker, ACS, CHF, hypertension, DM, dialysis, anemia, PAD, 15 ≤ eGFR < 30 ml/min/1.73 m^2^ vs. eGFR < 15 ml/min/1.73 m^2^, previous AMI, PCI, WBC, albumin, total Cholesterol, triglyceride, high-density lipoprotein cholesterol, Lg ProBNP, Lg D-dimer, ACEI/ARB, β-blockers, statins, diuretics

## Discussion

To our knowledge, this is the first study to evaluate the association between baseline LDL-C levels and long-term all-cause mortality in patients with CAD and AKD. Over a median follow-up period of 2.7 years, the overall incidence of all-cause death was 38.9% among patients with CAD and AKD at very high-risk in our study. Our results showed that the risk with respect to all-cause mortality was more pronounced among patients with baseline LDL-C level < 1.8 mmol/L. After adjusting for confounding factors, our data suggested that lower baseline LDL-C level (< 1.8 mmol/L) was significantly associated with higher long-term all-cause mortality among patients with CAD and AKD. Similar results were also observed in different subgroups.

Most previous studies referred to LDL-C focused on the relationship between LDL-C concentration after lipid-lowering therapy and clinical outcomes. Although, reduced LDL-C levels after lipid lowering therapy can improve the prognosis of CAD patients, but the effect would be weakened when renal function deteriorates. And, most trials involving patients with cardiovascular disease have either excluded patients with advanced kidney disease or included too few to permit a confident estimation of treatment benefits [19, 20]. Studies involving baseline LDL-C levels are limited.

Our study showed a negative relation between baseline LDL-C levels and long-term outcomes in patients with CAD and AKD. Similarly, a recent meta-analysis showed that benefits of lowering LDL-C were absent when the baseline LDL-C level was less than < 2.6 mmol/L [21]. Findings of an observational study conducted by Kovesdy et al. also showed that lower LDL cholesterol concentration was relevant to higher risk of mortality [11]. Furthermore, there were some previous studies investigating the inverse relations between baseline or admission LDL-C levels and clinical outcomes in the subgroups of coronary artery disease. Wang et al. demonstrated that lower admission LDL-C levels (< 2.6 mmol/L) were associated with better in-hospital survival in patients with acute coronary syndrome [22]. Cho et al. found that among patients with acute myocardial infarction, lower baseline LDL-C levels (< 1.8 mmol/L) were associated with higher mortality at 12 months [23]. Reddy et al. also found that in the AMI patients lower baseline LDL-C levels (< 2.0 mmol/L) were associated with higher in-hospital mortality [24]. According to Al-Mallah et al’s study, the relation of lower LDL-C levels (< 2.7 mmol/L) and poorer prognosis still existed [25]. There are noteworthy similarities between our study and the above four studies. Our study and the four studies all focused on CAD patients. The results of these studies, as well as ours, indicated the paradoxical phenomenon that low LDL-C levels are correlated to poor prognosis. The Findings from a study conducted by Hu et al. enrolling 3441 patients with CKD showed that no significant benefit was seen from statins in kidney function deterioration for patients with CKD [26]. While, for patients with baseline eGFR < 45 mL/min/1.73 m2 (CKD stage 3B-5), the effect of statins was significant. Among patients included in our study, renal function was all below 30 mL/min/1.73 m2. According to the conclusion of Hu et al.’s study, patients enrolled in our study would benefit from the use of statins. Furthermore, use of statins is important part of secondary prevention of coronary artery disease, and has been demonstrated to improve outcomes in patients with coronary heart disease. In our study, the use of statins can reduce the risk of long-term all-cause mortality by 43% (unadjusted HR: 0.63, 95% CI: 0.46–0.87). Thus, we included statins in the multivariate Cox model 6, in order to adjust for confounding effects of statins on prognosis.

There are several caveats to consider with regard to the interpretation of our results. First, in a previous study, an inverse association between LDL-C levels and incident cancer incidences was observed [27]. Although cardiovascular disease (CVD) is the leading cause of mortality among patients with CKD [28], some patients may die because of incident cancer during the follow-up period. Unfortunately, our follow-up data lack records of specific causes of death. Moreover, evidence is emerging that cholesterol is related to the regulation of immune cell function by improving their antitumor activity and activating immune signaling, which may provide novel insights into the role of cholesterol in the development of cancer [29–31]. Second, a plausible explanation for the absence of a positive correlation between LDL-C and long-term all-cause mortality is that patients with CAD and AKD in the low LDL-C group were in poor condition and had many complications. Patients with low LDL-C concentrations (< 1.8 mmol/L) had higher prevalence of cardiovascular disease related comorbidities and other diseases or conditions associated with prognosis, including diabetes mellitus (62.3% vs. 51.7%), hypertension (89.9% vs. 79.7%), anemia (91.3% vs. 81.0%) and poor renal function. Similarly, in Kovesdy et al.’s study, the paradoxical relationship between lower LDL-C levels and higher risk of death attenuated as the adjustment variables increased. After further consideration of baseline confounders, particularly malnutrition-inflammation-cachexia syndrome, lower LDL-C concentration was no longer independently relevant to increased risk of mortality [11]. Furthermore, according to the result of our subgroup analysis, patients who were on dialysis and suffering from diabetes mellitus also had a higher risk of death when LDL-C < 1.8 mmol/L. The increased long-term mortality may result from basic diseases to some extent.

According to our results of the association between low baseline LDL-C levels and increased long-term all-cause mortality in patients with CAD and AKD, our data may provide clinical evidence for the management of LDL-C in this population. First, in this very high-risk population, low baseline LCL-C level may be a reflection of patients with multiple diseases and adverse conditions. In the long-term management of patients with CAD and AKD, excessively low LDL-C may provide a new risk assessment indicator for clinical decisions. Second, patients with LDL-C at very low levels should be carefully assessed and if necessary, screened for other diseases that may affect mortality. On the other hand, during the treatment of high LDL-C, regular follow-up is required to dynamically evaluate the lipid levels and adjust the lipid-lowering strategies. Ultimately, new multicenter prospective or RCT clinical studies for baseline LDL-C management in these patients should be conducted to evaluate the appropriate lipid management strategy.

### Limitation

This study has several limitations. First, this study was a retrospective single-center study conducted at Guangdong Cardiovascular Institute, Guangdong Provincial People’s Hospital in China. However, the sample size of the study was relatively large; thus, this study can be the basis for further prospective RCTs. Second, in this study, we used only baseline data of LDL-C, and we lacked LDL-C data during the follow-up period. The long-term prognosis correlation may be affected by treatment such as statins, kidney function and other factors. However, we corrected for mixed factors such as dialysis dependence, statin treatment when discharged, age, and other risk factors. Third, there was limited data for the included patients, without information about BMI which might help us assess nutritional status. However, we chose the serum albumin concentration as the measure of nutritional status. Ultimately, the outcome of the study was defined as all-cause death, and some specific reasons may not be clear. However, cardiovascular death was the leading cause of death in these patients.

## Conclusion

This study is the first to find that among very high-risk patients with CAD and AKD, a lower baseline LDL-C level (< 1.8 mmol/L) did not predict a higher survival rate but was associated with a worse prognosis. Our findings suggested that LDL-C may not be “the lower, the better” among all patients with CAD, and may even harm those with CAD combined with AKD. These findings suggest an awareness of striking a balance to avoid LDL cholesterol levels that are too low for mortality and need verification in further randomized clinical trials among patients with CAD and AKD.

## Supplementary Information

**Additional file 1: Supplementary Table S1**. Descriptive statistics among completed and missing data set. **Supplementary Table S2**. Univariable Cox regression analysis of long-term all-cause mortality.

## Data Availability

Not applicable at this stage. The datasets analyzed during the current study will be available from the corresponding author on reasonable request when the study is finished.

## Abbreviations

LDL-C: Low-density lipoprotein cholesterol
CAD: Coronary artery disease
AKD: Advanced kidney disease
CAG: Coronary angiography
PCI: Percutaneous coronary intervention
CHF: Congestive heart failure
HR: Hazard ratio
CI: Confidence interval
PAD: Peripheral arterial disease
AMI: Previous acute myocardial infarction
DM: Diabetes mellitus
