# Supplementary material for: Association between baseline LDL-C and prognosis among patients with coronary artery disease and advanced kidney disease
Source: BMC Nephrol. 2021 May 6;22:168. doi: 10.1186/s12882-021-02375-1 (PMC8101096; doi:10.1186/s12882-021-02375-1)
Supplement: Supplementary file 1 — Additional file 1: Supplementary Table S1. Descriptive statistics among completed and missing data set. Supplementary Table S2. Univariable Cox regression analysis of long-term all-cause mortality. [file 12882_2021_2375_MOESM1_ESM.docx]

**Association Between Baseline LDL-C and Prognosis among Patients with Coronary Artery Disease and Advanced Kidney Disease**

Bo Wang, MD ^a§^, Shiqun Chen, MS ^a§^, Jin Liu, MD ^a§^, Yan Liang, MD ^b^, Liangguang Meng, MD ^a^, Xiaoming Yan, MD ^c^, Haozhang Huang, MD ^d^, Guanzhong Chen, MD ^e^, Zhidong Huang, BS ^a^, Danyuan Xu, MS ^a^, Min Li, BS ^a^, Jingjing Liang, MD ^d^, Shuangxin Liu, MD, PhD ^f^, Jiyan Chen, MD, PhD, FACC, FESC ^a,d,e^, Yong Liu, MD, PhD, FACC ^a,d,e^*, Ning Tan, MD, PhD, FACC, FAPSIC, FESC ^a,d,e^*

^a^ Department of Cardiology, Guangdong Provincial Key Laboratory of Coronary Heart Disease Prevention, Guangdong Cardiovascular Institute, Guangdong Provincial People’s Hospital, Guangdong Academy of Medical Sciences, Guangzhou, 510080, China

^b^ Maoming People's Hospital, Maoming, 525000, China

^c^ Department of Information Technology, Guangdong Provincial People’s Hospital, Guangdong Academy of Medical Sciences, Guangzhou, 510080, China

^d^ The Second School of Clinical Medicine, Southern Medical University, Guangzhou, 510515, China

^e^ Guangdong Provincial People’s Hospital, School of Medicine, South China University of Technology, Guangzhou, 510100, China

^f^ Division of Nephrology, Guangdong Provincial People’s Hospital, Guangdong Academy of Medical Sciences, Guangzhou, 510080, China

^§^Contributed equally

*Address for correspondence

Ning Tan, MD, PhD, FACC, FAPSIC, FESC; Yong Liu, MD, PhD, FACC.

Department of Cardiology, Guangdong Provincial Key Laboratory of Coronary Disease, Guangdong Cardiovascular Institute, Guangdong Provincial People’s Hospital, South China University of Technology, Southern Medical University, Guangzhou 510080, China.

Tel: (+86) 02083827812-10528/Fax: (+86) 02083851483

Email: tanning100@126.com; liuyong@gdph.org.cn

**Supplementary Table S1.** Descriptive statistics among completed and missing data set

| Characteristic | Complete  (N=655) | Missing  (N=148) | P value |
| --- | --- | --- | --- |
| **Demographic characteristics** | | | |
| Age, year | 67.6 (10.3) | 66.6 (10.3) | 0.30 |
| Male, n (%) | 443 (67.6) | 107 (72.3) | 0.32 |
| **Coexisting conditions** | | | |
| Current smoker, n (%) | 97 (14.8) | 20 (13.5) | 0.78 |
| ACS, n (%) | 300 (45.8) | 86 (58.1) | 0.01 |
| CHF, n (%) | 328 (50.1) | 53 (35.8) | 0.002 |
| Hypertension, n (%) | 531 (81.1) | 123 (83.1) | 0.65 |
| Diabetes mellitus, n (%) | 354 (54.0) | 75 (50.7) | 0.52 |
| Dialysis, n (%) | 237 (36.2) | 46 (31.1) | 0.28 |
| Anemia, n (%) | 543 (82.9) | 119 (81.5) | 0.78 |
| PAD, n (%) | 72 (11.0) | 19 (12.8) | 0.62 |
| eGFR, ml/min/1.73 m^2^ | 17.7 (8.2) | 19.1 (8.7) | 0.08 |
| 15≤ eGFR < 30 ml/min/1.73 m^2^, n (%) | 401 (61.2) | 98 (66.2) | 0.30 |
| Previous AMI, n (%) | 56 (8.5) | 8 (5.4) | 0.27 |
| PCI, n (%) | 352 (53.7) | 88 (59.5) | 0.24 |
| **Laboratory examination** | | | |
| WBC, 10^9^/L | 8.6 (3.3) | 8.4 (3.4) | 0.45 |
| ALB, g/L | 32.9 (5.0) | 32.1 (5.7) | 0.10 |
| Total Cholesterol, mmol/L | 4.4 (1.3) | 4.4 (1.2) | 0.99 |
| LDLC, mmol/L | 2.7 (1.0) | 2.6 (0.9) | 0.21 |
| TRIG, mmol/L | 1.9 (1.4) | 1.9 (1.4) | 0.95 |
| HDLC, mmol/L | 0.9 (0.3) | 0.9 (0.3) | 0.69 |
| Lg ProBNP, pg/ml | 3.8 (0.7) | 3.7 (0.6) | 0.52 |
| Lg D-dimer, ng/mL | 3.1 (0.4) | 3.0 (0.4) | 0.40 |
| **Medications** | | | |
| ACEI/ARB, n (%) | 272 (41.5) | 57 (38.5) | 0.56 |
| β-blockers, n (%) | 436 (66.6) | 106 (71.6) | 0.28 |
| Statins, n (%) | 587 (89.6) | 130 (87.8) | 0.63 |
| Diuretics, n (%) | 289 (44.1) | 48 (32.4) | 0.01 |
| **Events** | | | |
| Death, n (%) | 257 (39.2) | 55 (37.2) | 0.71 |

* Data are presented as the mean value standard deviation or percentage of participants.

Abbreviations: LDL-C, low-density lipoprotein cholesterol; ACS, acute coronary syndrome; CHF, congestive heart failure; PAD, peripheral arterial disease; eGFR, estimated glomerular filtration rate; AMI, acute myocardial infarction; PCI, percutaneous coronary intervention; HDL-C, high-density lipoprotein cholesterol; TRIG, triglycerides; WBC, white blood cell; pro-BNP, pro-brain natriuretic peptide; ACEI, Angiotensin-Converting Enzyme Inhibitors; ARB, Angiotensin Receptor Blockers.

**Supplementary Table S2.** Univariable Cox regression analysis of long-term all-cause mortality

| Characteristics | Univariate | |  | |
| --- | --- | --- | --- | --- |
|  | HR | 95% CI | | P value |
| LDL-C < 1.8mmol/L  vs. LDL-C ≥ 1.8mmol/L | 1.53 | 1.18 | 1.99 | 0.001 |
| Age, year | 1.01 | 0.99 | 1.02 | 0.13 |
| Male | 1.35 | 1.05 | 1.73 | 0.02 |
| Current smoker | 1.08 | 0.80 | 1.48 | 0.61 |
| ACS | 1.00 | 0.80 | 1.25 | 0.99 |
| CHF | 1.54 | 1.23 | 1.93 | < 0.001 |
| Hypertension | 1.06 | 0.79 | 1.43 | 0.70 |
| Diabetes mellitus | 1.14 | 0.91 | 1.43 | 0.24 |
| Dialysis | 1.33 | 1.06 | 1.67 | 0.01 |
| Anemia | 1.75 | 1.25 | 2.45 | 0.001 |
| PAD | 0.90 | 0.63 | 1.29 | 0.57 |
| 15 ≤ eGFR < 30 ml/min/1.73 m^2^  vs. eGFR < 15 ml/min/1.73 m^2^ | 0.60 | 0.48 | 0.75 | < 0.001 |
| Previous AMI | 1.40 | 0.97 | 2.03 | 0.07 |
| PCI | 0.95 | 0.76 | 1.19 | 0.66 |
| WBC, 10^9^/L | 1.01 | 0.98 | 1.04 | 0.51 |
| ALB, g/L | 0.96 | 0.94 | 0.98 | < 0.001 |
| Total Cholesterol, mmol/L | 0.94 | 0.87 | 1.03 | 0.20 |
| TRIG, mmol/L | 0.99 | 0.91 | 1.07 | 0.74 |
| HDLC, mmol/L | 0.79 | 0.51 | 1.23 | 0.30 |
| Lg ProBNP, pg/ml | 1.75 | 1.46 | 2.09 | < 0.001 |
| Lg D-dimer, ng/mL | 1.84 | 1.41 | 2.39 | < 0.001 |
| ACEI/ARB | 0.89 | 0.71 | 1.12 | 0.32 |
| β-blockers | 0.60 | 0.48 | 0.76 | < 0.001 |
| Statins | 0.63 | 0.46 | 0.87 | 0.005 |
| Diuretics | 1.24 | 0.99 | 1.54 | 0.06 |

Abbreviations: LDL-C, low-density lipoprotein cholesterol; ACS, acute coronary syndrome; CHF, congestive heart failure; PAD, peripheral arterial disease; eGFR, estimated glomerular filtration rate; AMI, acute myocardial infarction; PCI, percutaneous coronary intervention; WBC, white blood cell; ALB, albumin; TRIG, triglycerides; HDL-C, high-density lipoprotein cholesterol; pro-BNP, pro-brain natriuretic peptide; ACEI, Angiotensin-Converting Enzyme Inhibitors; ARB, Angiotensin Receptor Blockers.
